# pycmplot: fast, low-memory multi-track circular and linear Manhattan plotting in Python

**DOI:** 10.1093/bioadv/vbag281

**Published:** 2026-09-22

**Authors:** Kevin Esoh, Fujr Osman, Cesar Fortes-Lima, Gordon A Awandare, Ambroise Wonkam

**Affiliations:** McKusick-Nathans Institute and Department of Genetic Medicine, Johns Hopkins University School of Medicine, Baltimore MD 21205, United States; West African Centre for Cell Biology of Infectious Pathogens (WACCBIP), College of Basic and Applied Sciences, University of Ghana, Legon, Box LG 54, Ghana; Division of Human Genetics, Faculty of Health Sciences, University of Cape Town, Cape Town, Observatory 7925, South Africa; McKusick-Nathans Institute and Department of Genetic Medicine, Johns Hopkins University School of Medicine, Baltimore MD 21205, United States; McKusick-Nathans Institute and Department of Genetic Medicine, Johns Hopkins University School of Medicine, Baltimore MD 21205, United States; Department of Organismal Biology, Evolutionary Biology Centre, Human Evolution Program, Uppsala University, Box 256, SE-751 05 Uppsala, Sweden; West African Centre for Cell Biology of Infectious Pathogens (WACCBIP), College of Basic and Applied Sciences, University of Ghana, Legon, Box LG 54, Ghana; McKusick-Nathans Institute and Department of Genetic Medicine, Johns Hopkins University School of Medicine, Baltimore MD 21205, United States

## Abstract

**Motivation:**

Manhattan plots remain the standard visual summary for genome-wide association studies and other per-variant scans (iHS, FST, XP-EHH). Existing Manhattan visualization tools either lack multi-track support (gwaslab, qmplot, and qqman) or do not provide a Circos-style multi-track circular Manhattan view. CMplot (R) does, but its memory and runtime costs on multi-million-variant inputs are substantial.

**Results:**

We present pycmplot, a Python package for generating multi-track linear and Circos-style circular Manhattan plots from one or more GWAS summary statistics. On a 10-million-variant scan, pycmplot is 5.5× faster and uses ∼5× less memory than CMplot for linear Manhattan plotting, 1.6× faster (and 3× faster with optional pre-filtering) for circular Manhattan, and 7.7× faster for qq rendering. Multi-track comparisons of two summary statistics are 4.5× faster (linear) and 3.5× faster (circular) than CMplot’s equivalent mode. Transparent hg18/hg19 → hg38 liftover lets users mix coordinate systems within a single plot, and a lightweight bundle of Ensembl GFF3 gene information files (hg19 and hg38) enables nearest gene annotation.

**Availability and implementation:**

pycmplot is freely available under the MIT license at https://github.com/esohkevin/pycmplot; and from PyPI (pip install pycmplot). Documentation is at https://pycmplot.readthedocs.io.

## 1 Introduction

Manhattan plots have been the canonical visualization of GWAS results since the study by Klein *et al*. (2005), and Circos-style circular variants (Krzywinski *et al*. 2009) have become a popular space-efficient way to display whole-genome scans. As reference panels have grown—TOPMed Freeze 8 (Taliun *et al*. 2021) imputes to ∼308 million variants; 1000 Genomes 30× WGS (Byrska-Bishop *et al*. 2022) yields ∼70 million–a single Manhattan plot can routinely require 10–100 million scatter points to be rendered. At those scales, the choice of plotting tool becomes a major consideration in terms of turnaround time and the cluster memory footprint, particularly when several traits or ancestry-stratified scans are compared side by side.

Two of the most widely used dedicated tools are written in R: CMplot (Yin *et al*. 2021), which supports multi-track linear and circular Manhattan and quantile-quantile (qq) plots in one package, and qqman (Turner 2018), a lightweight single-track linear and qq plotter. The Python ecosystem currently offers gwaslab (He *et al*. 2023) and qmplot (https://github.com/ShujiaHuang/qmplot; accessed 2026), both of which are competitive on single-track linear Manhattan/qq but do not natively support a Circos-style multi-track circular Manhattan view comparable to CMplot. pycmplot fills that gap with a Python-native, matplotlib-based implementation that scales to multi-million-variant inputs while remaining the fastest and lowest-memory of the tools we compared.

## 2 Implementation

### 2.1 Multi-track circular Manhattan plotting

The main feature is a multi-track Circos-style circular Manhattan view (Fig. 1). Each input summary-statistics file becomes a concentric track; chromosomes are sectors of the outermost ring shared by all tracks; lead SNPs and a structured hits table are produced for every track and used to annotate the plot (a hits table with lead SNPs can also be user-supplied in the python API, directly allowing for customizable annotations). The circular layout is built on top of pycirclize (https://github.com/moshi4/pyCirclize; accessed 2026) with custom positioning logic that keeps annotation labels off the data, supports gene-name labelling resolved from the bundled Ensembl (Herrero *et al*. 2016) GFF3 tables, and harmonizes sector sizes across tracks so chromosomes line up radially. The same multi-track machinery drives the linear Manhattan plotter, so users can switch between the two views by setting a single command line interface (CLI) flag (—mode cm | lm).

**Figure 1:**
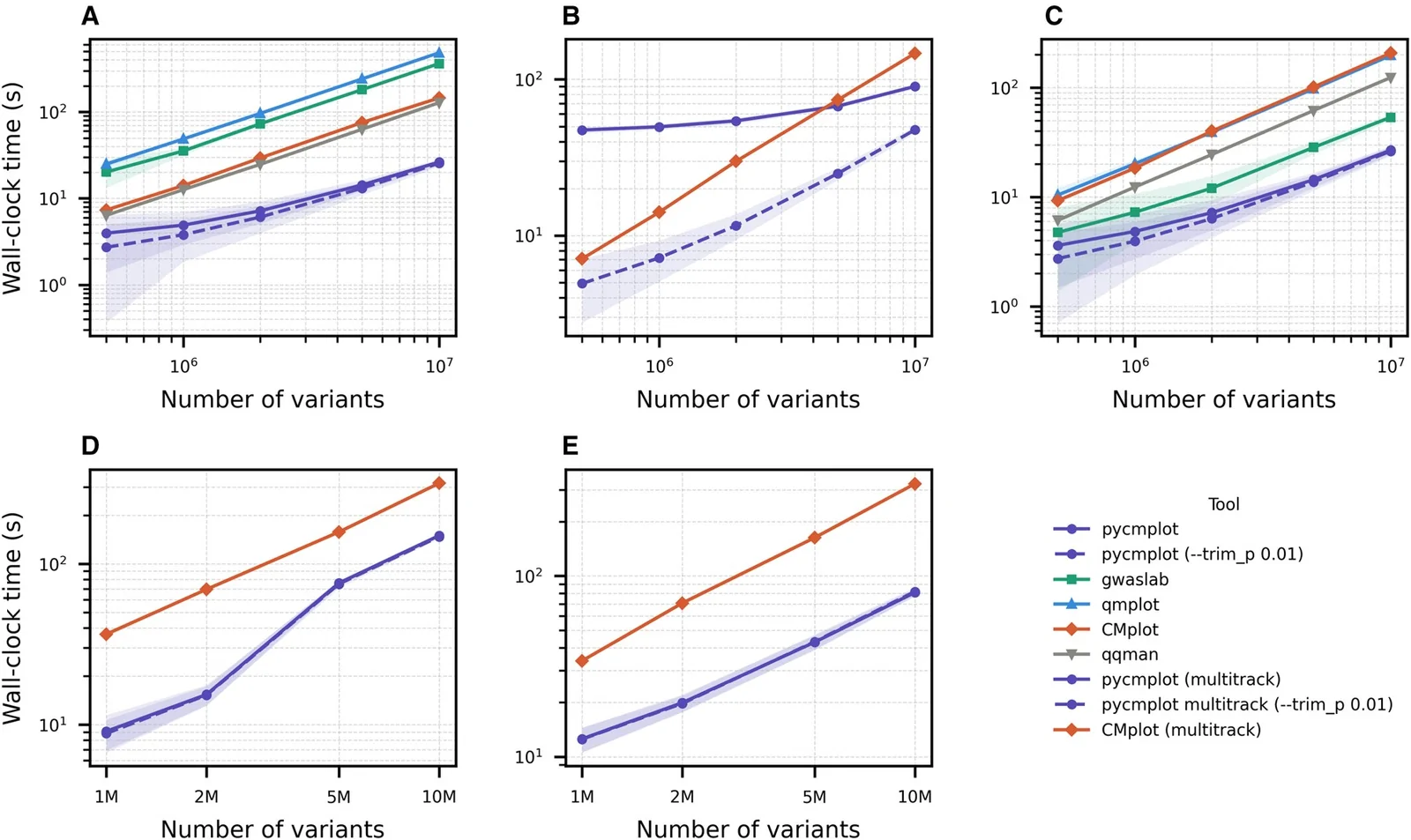
Wall-clock runtime scaling, log–log axes, shaded bands = ±1 s.d. across 5 replicates. Top row, single-track scans (500K–10M variants): (A) Linear Manhattan–pycmplot is 5.5× faster than CMplot and 4.9× faster than qqman at 10M variants. (B) Circular Manhattan–only CMplot offers a comparable circular mode; pycmplot crosses below CMplot at ∼5M variants and the—trim_pval 0.01 variant is 3× faster than CMplot at 10M. (C) qq–pycmplot is the fastest of all tools at every size. Bottom row, multi-track scans of three-trait summary statistics with (Trait 1, Trait 2, and Trait 3) at four variants count simulations (1M, 2M, 5M, and 10M variants): (D) Multi-track linear Manhattan and (E) Multi-track circular Manhattan–pycmplot is 4.5× and 3.5× faster than the equivalent CMplot multi-track mode at 10M variants, with the—trim_pval 0.01 variant essentially overlapping the untrimmed pycmplot line (most of the work has already been done by default auto-thinning). The multi-track comparison is restricted to pycmplot and CMplot because neither gwaslab, qmplot nor qqman supports a native multi-track Manhattan view.

### 2.2 Performance and density-aware sub-sampling

pycmplot keeps memory and time low through three complementary mechanisms. First, a default-on density-aware sub-sampling step [inspired by gwaslab (He *et al*. 2023)] preserves every variant whose − log10(P) is above a user-configurable threshold (default 2.0, i.e. *P* ≤ .01) and uniformly down-samples the dense null background to 200 000 rows per track at most. For non-*P*-value statistics (iHS, XP-EHH, FST, Fay & Wu’s H, and Tajima’s *D*), the same machinery operates on |statistic|, so suggestive and outlier signals at both tails of signed distributions are preserved verbatim. Second, scatter rendering uses matplotlib.axes.Axes.plot (Line2D) per chromosome rather than the standard scatter (PathCollection). Line2D uses a default-on feature called should_simplify to check points with redundant coordinates that can be safely excluded without altering visualization thus reducing draw time by ∼10× for multi-million-variant tracks. Third, the loader uses the pyarrow CSV engine where available, stores chromosome labels as an ordered Categorical (so normalization runs once over ∼25 distinct values rather than per row), and skips constructing the per-track *P*-value array when no qq plot is requested via the—qq flag.

An additional—trim_pval option layers a hard *P*-value pre-filter on top of auto-thinning for users who want to discard everything below a suggestive threshold up front. Combining the two cuts the 10-million-variant circular Manhattan rendering time from ∼38 minutes (CMplot) to ∼47 s (pycmplot).

### 2.3 Mixed genome coordinate systems

Trans-ancestry meta-analyses and re-imputation pipelines often produce summary statistics in mixed genome builds (hg18, hg19, hg38). Manually harmonizing these before plotting is error prone. pycmplot accepts a “BUILD” column (or a per-file build supplied via—build) on the input and lifts hg18 or hg19 coordinates to hg38 on the fly using bundled UCSC chain files via pyliftover (https://github.com/konstantint/pyliftover; accessed 2026). Only rows whose build is hg18 or hg19 are touched; rows already in hg38 are passed through unchanged. The result is that several summary statistics in different build coordinates can be concatenated into a single file (or supplied as separate files with per-file “—build” option), and plotting proceeds in a unified hg38 coordinate system without preprocessing. Mixed build and hg18-only summary statistics are harmonized to hg38 and a bundled lightweight version of Ensembl GFF3 file in hg38 is used for gene annotation. If the summary statistics are in hg19 only, liftover is not performed and a bundled Ensembl GFF3 file in hg19 is used for annotation. To our knowledge, no other Python or R Manhattan-plot tool offers this level of clear coordinate harmonization. The procedure for gene selection for annotation used by pycmplot is detailed in the Supplementary Material, section S8 and Fig. S1.

### 2.4 Interface and workflow integration

pycmplot ships with a unified command-line interface and an equivalent Python API. Column names (“CHR”, “POS”, “SNP”, “P”, and “BUILD”) are auto-detected from common naming conventions but can be specified explicitly. Output is rasterized at the matplotlib backend level so PDF and SVG output files remain compact even for multi-million-variant inputs (typical Manhattan PDF: 50 KB at 10M variants). The package depends only on standard scientific-Python libraries (numpy, pandas, matplotlib, scipy, pycirclize, pyliftover, and natsort), is tested against Python 3.9–3.13, and is published on PyPI (see the Availability statement).

## 3 Benchmarking

We benchmarked pycmplot against gwaslab, qmplot, CMplot and qqman on synthetic GWAS summary statistics of 500k, 1M, 2M, 5M and 10M variants (Methods in Supplement; data generated with bundled generate_sumstats.py). All runs were single-core (OMP_NUM_THREADS = 1, MKL_NUM_THREADS = 1) with 64 GB memory, 5 replicate timings each, no thread pinning across tools. Wall-clock time was measured with time.perf_counter and peak memory with tracemalloc (Python tools) and/usr/bin/time -v (R tools).

Figure 1 shows the runtime scaling for all five plot types. pycmplot has the shallowest growth across the three single-track scans (Fig. 1A–C); at 10 million variants it is the fastest tool for qq [26.8 s vs 53.6 s for gwaslab (He *et al*. 2023), the next fastest] and matches or beats both R tools for linear Manhattan [26.3 s vs 128.0 s qqman (Turner 2018), 145.1 s CMplot]. For circular Manhattan–where the pycirclize back-end carries some per-track overhead at small N—pycmplot crosses below CMplot between 2 and 5 million variants; with the optional—trim_pval 0.01 filter pycmplot is strictly the fastest at every size tested. The advantage is largest in multi-track mode (Fig. 1D–E), the typical use case: at 10 M variants per track, pycmplot completes the two-track linear Manhattan in 149.8 s vs 316.8 s for CMplot (2.1×) and the three-track circular Manhattan in 81.0 s vs 323.4 s (4×). No other Python tool in our comparison offers a native multi-track Manhattan mode. Table 1 summarizes the 10 M-variant single-track and multi-track timings and peak memory for single-track and multi-track visualizations. At 10 M variants, pycmplot uses 2 GB vs 8.2 GB for CMplot for single-track, and 1.7 GB vs 9.4 GB for CMplot for multi-track. pycmplot had the smallest peak memory of all tools tested. The complete benchmark CSVs, the rendered output of each tool, and reproducible launch scripts are in the package repository under ‘benchmark’.

**Table 1:** Wall-clock time at 10 M variants for single-track and multi-track benchmarks; mean ± 1 s.d. across 5 replicates.

Tool	Manhattan (s)	Circular (s)	qq (s)	**Peak memory (MB)** *
Single-track				
pycmplot	26.3 ± 2.1	90.2 ± 2.3	26.8 ± 2.2	1514
pycmplot (—trim_pval 0.01)	25.5 ± 2.1	47.5 ± 2.3	26.3 ± 2.3	1514
gwaslab	362.8 ± 4.4	–	53.6 ± 3.4	6039
qmplot	483.9 ± 1.5	–	196.7 ± 2.5	4678
CMplot	145.1 ± 0.9	146.7 ± 1.7	206.8 ± 2.2	8172
qqman	128.0 ± 0.5	–	123.6 ± 0.2	2641
Multi-track				
pycmplot	149.8 ± 3.6	81.0 ± 3.8	–	1669
pycmplot (—trim_pval 0.01)	147.5 ± 3.9	82.0 ± 4.4	–	1669
CMplot	316.8 ± 1.7	323.4 ± 4.0	–	9442

– = mode not supported by tool.

*Peak memory shown for linear Manhattan at 10 M variants (single-track) and circular Manhattan at 10 M variants (multi-track).

Since its initial release, pycmplot (v0.4.0) has been extended to improve the efficiency and flexibility of large-scale, iterative visualization workflows. A per-track caching layer uses a SHA-256 hash of each input file and its Stage-1 processing parameters to identify reusable results, allowing unchanged tracks to be loaded directly from lightweight cache during subsequent runs while automatically invalidating tracks affected by parameter changes. The automatically generated hits summary is now also saved as a user-editable, group-scoped table, through which users can assign custom colors and categories to loci; these annotations are propagated across linear and circular plots and preserved across cache regeneration based on genomic coordinates. Additional improvements include optional *P*-value computation for QQ plots to reduce memory usage and safer handling of multi-panel rendering. These changes substantially improve the performance, customization, and reproducibility of repeated plotting workflows while reducing the computational burden associated with large summary-statistics datasets. We note that the performance of the Stage-1 data loading and preparation step remains unchanged as from pycmplot v0.2.8 that was used in the initial benchmark of the tools. None of the other tools we compared pycmplot to had the additional features.

## 4 Example usage with real summary statistics

We demonstrate scenarios where pycmplot visualization could be useful by generating plots for multi-panel genome-wide association studies (GWAS) that we have recently conducted including GWAS of fetal hemoglobin (HbF) in individuals with sickle cell disease (SCD) of African ancestry (Wonkam *et al*. 2025). This study involved the utilization of multiple reference haplotype panels for genotype imputation and association analysis using data from all panels, rather than selecting a “best-performing” panel. Figure 2 shows significant signals observed in specific panels or shared across the panels represented compactly in one figure, allowing direct visual comparison. The same summary statistics were plotted four times to demonstrate different features. The plot was generated using the Python API specifically to demonstrate the multi-panel canvas feature (see code in the Supplementary Material). Demonstrations of the behavior of the ‘—build’ feature using the command line interface (CLI) are displayed in the Supplementary Material Figs. S2–S5. Demonstrations of signed statistics–such as integrated haplotype score commonly calculated in selection scans–are shown in Figs. S6 and S7. The commands used to generate the figures are also shown. A tutorial detailing other use cases and the behavior of key features is available at https://pycmplot.readthedocs.io/en/latest/cli_tutorial.html.

**Figure 2:**
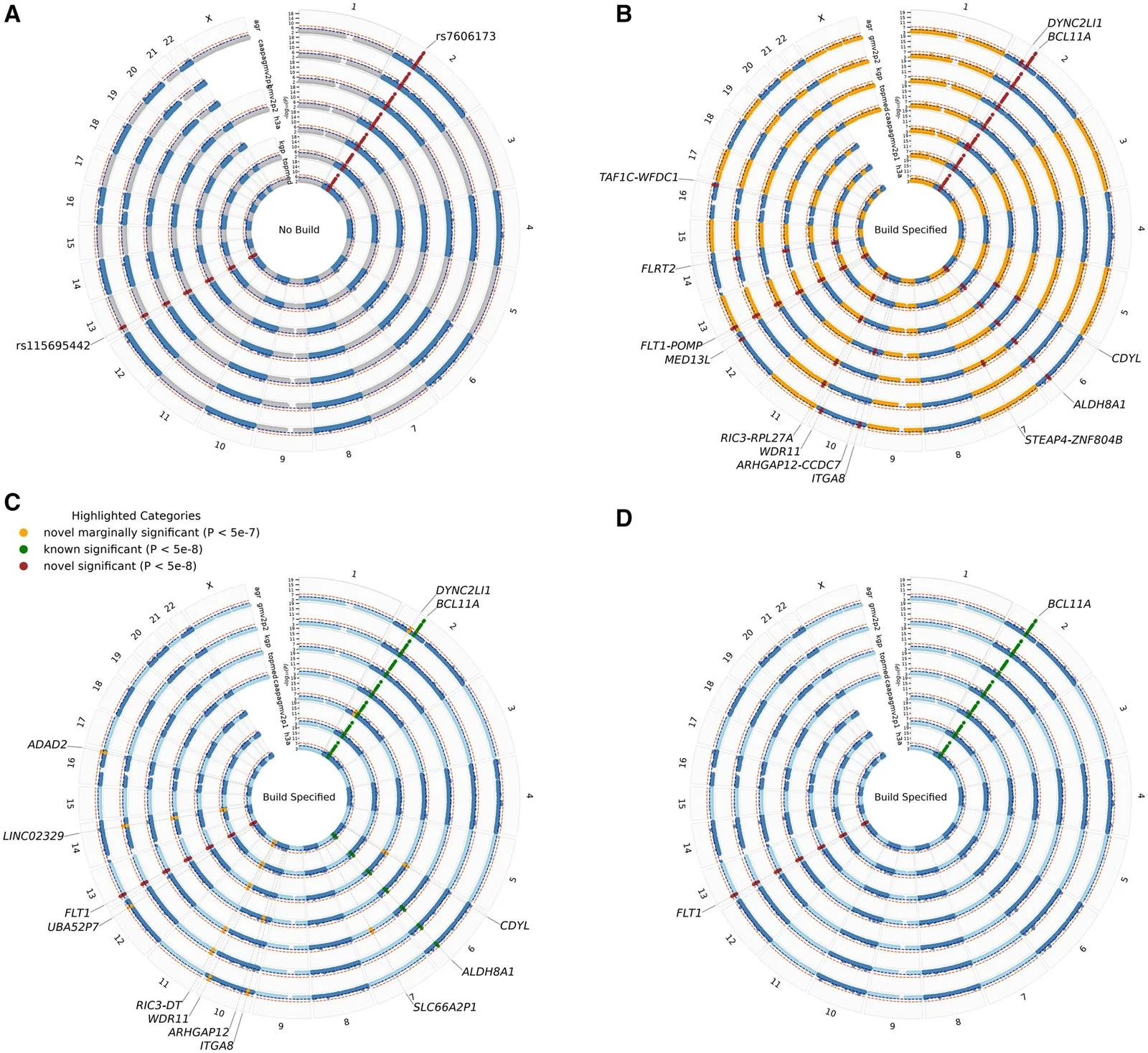
Circular multi-track Manhattan plots of HbF GWAS in SCD in Cameroon. Summary statistics from the published study by Wonkam *et al*. (2025) were used. pycmplot now generates a hits overlay table that can be edited to customize plots with different color categories, including adding a legend. (A) when no genome build information is specified, pycmplot safely annotates by “SNP” to avoid wrong gene annotations in case of mixed genomic coordinates, (B) genome build is specified and signals annotated by “top_gene” from the unedited hits overlay table, including annotating sub-genome-wide significant signals and changing the alternate chromosome colors, (C) the hits overlay table is edited with specific categories that are annotated by “nearest_gene” and highlighted, (D) only significant categories are highlighted and annotated. A single legend at (C) is shared with (D). Users can also choose to include separate legends.

## 5 Conclusion

pycmplot brings Circos-style multi-track Manhattan plotting to the Python GWAS ecosystem, with performance and memory characteristics that are competitive at modest scan sizes and substantially better at the 10-million-variant scale that is now routine. Default-on density-aware sub-sampling, transparent hg18/hg19 → hg38 liftover, and a unified CLI/API make it a drop-in option for analysts who already build their downstream pipelines in Python. Active development is at the GitHub repository; future releases will extend support to functional and LD-aware annotation, configurable LD reference panels, Telomere-to-Telomere (T2T-CHM13) coordinates as they become standard, and annotation for other species.

## Supplementary Material

vbag281_Supplementary_Data

## Data Availability

Source code, documentation, benchmark scripts, and a Jupyter Notebook step-by-step tutorial of the python API are available in GitHub at https://github.com/esohkevin/pycmplot. Released versions are available on PyPI at https://pypi.org/project/pycmplot/ and can be installed using ‘pip install pycmplot’. Documentation is provided via Read The Docs at https://pycmplot.readthedocs.io. Licence: MIT. All summary statistics files used in the benchmark were simulated and can be generated using the “generate_sumstats.py” and “generate_multi_sumstats.py” scripts in the benchmark folder of the Github repository.

## Use of AI tools

During development of pycmplot, we used Anthropic’s Claude (Sonnet 4.6 and Opus 4.8; large language model) as a coding assistant. Specific contributions of the Artificial Intelligence (AI) assistant included: refactoring sections of the Python source (notably the auto-thinning, multi-rail annotation layout, and rendering paths in pycmplot.plotting.linear), profiling the untrimmed plotting pipeline with cProfile and proposing the optimizations subsequently incorporated, drafting the benchmark aggregation script (benchmark/collect_results.py) and the composite figure builder (benchmark/build_composite_figure.py), and copy-editing the present manuscript. All scientific decisions–choice of benchmark design, tools to compare against, threshold and parameter defaults, claims made in the text–were ours (the authors). We reviewed every AI-generated change before incorporation, and we take full responsibility for the correctness and integrity of the work.
