# Supplementary material for: pycmplot: fast, low-memory multi-track circular and linear Manhattan plotting in Python
Source: Bioinform Adv. 2026 Sep 22;6(1):vbag281. doi: 10.1093/bioadv/vbag281 (PMC13641829; doi:10.1093/bioadv/vbag281)
Supplement: Supplementary file 1 [file vbag281_supplementary_data.pdf]

Supplementary Methods: Performance Benchmarking

pycmplot: fast, low-memory multi-track circular and linear Manhattan plotting in Python

S1. Overview

We benchmarked pycmplot against four established GWAS visualization tools: *CMplot* (R) (Yin *et al.* 2021), *gwaslab* (Python) (He *et al.* 2023), *qmplot* (Python; https://github.com/ShujiaHuang/qmplot; accessed 2026) , and *qqman* (R) (D. Turner 2018). Benchmarks were conducted across a range of dataset sizes on a high-performance computing (HPC) cluster, reflecting the primary deployment contexts for GWAS visualization tools. Single-trait and multi-trait (multi-track) plotting were benchmarked separately, as these represent distinct use cases with fundamentally different input format requirements across tools.

S2. Tools Evaluated

Table S1 summarizes the tools included in this benchmark. For each tool, the installed version, implementation language, and supported plot types benchmarked are listed. All tools were installed in dedicated conda environments to ensure version isolation (see S5.2).

Table S1. GWAS visualization tools included in this benchmark.

Tool	Language	Version	Plot types benchmarked	Source
pycmplot	Python	0.2.8	Manhattan (linear), Manhattan (circular), QQ, multi-track Manhattan, multi-track circular	PyPI; GitHub
CMplot	R	4.5.1	Manhattan (linear), Manhattan (circular), QQ, multi-track Manhattan, multi-track circular	CRAN; GitHub
gwaslab	Python	4.1.6	Manhattan (linear), QQ	PyPI; GitHub
qmplot	Python	0.3.3	Manhattan (linear), QQ	PyPI; GitHub
qqman	R	0.1.9	Manhattan (linear), QQ	CRAN

S3. Benchmark Datasets

S3.1 Single-trait datasets

Synthetic GWAS summary statistics were generated at five variant counts (Table S2) using the provided *generate_sumstats.py* script. Variants were distributed across chromosomes 1–22 proportionally to approximate GRCh38 chromosome lengths, with positions drawn uniformly within chromosome boundaries. Summary statistics included: chromosome, position, SNP identifier, reference and alternate alleles, effect size (β), standard error, and p-value. Background p-values were drawn from a uniform distribution U(0, 1); 30 association signals were injected at randomly selected positions with p-values drawn from log-uniform(10−50, 10−8). All datasets were generated with a fixed random seed (seed = 42) for reproducibility.

Table S2. Single-trait benchmark dataset sizes.

Label	Variants (N)	Approx. size (TSV)	Notes
500K	500,000	~75 MB	All tools; HPC
1M	1,000,000	~150 MB	All tools; HPC
2M	2,000,000	~300 MB	All tools; HPC
5M	5,000,000	~750 MB	All tools; HPC
10M	10,000,000	~1.5 GB	All tools; HPC

S3.2 Multi-trait datasets

For multi-track benchmarks, three-trait summary statistics were generated at four variant counts using *generate_multi_sumstats.py* (Table S3). All traits shared an identical variant scaffold (chromosome, position, SNP identifiers, and alleles) generated with seed = 42, reflecting a realistic scenario in which multiple phenotypes are measured in the same cohort. Independent p-values and effect sizes were generated for each trait using distinct seeds (Trait 1: seed = 101, Trait 2: seed = 102, Trait 3: seed = 103). The 10M dataset was excluded from multi-trait benchmarks due to memory constraints on the laptop environment and the impracticality of the 30 GB combined file size on HPC scratch storage.

Table S3. Multi-trait benchmark dataset sizes (three traits per size).

Label	Variants per trait	Total size (3 files)	Notes
1M	1,000,000	~450 MB	pycmplot vs CMplot; HPC
2M	2,000,000	~900 MB	pycmplot vs CMplot; HPC
5M	5,000,000	~1.3 GB	pycmplot vs CMplot; HPC
10M	10,000,000	~2.5 GB	pycmplot vs CMplot; HPC

Trait files were saved as separate TSV files and referenced via a plain-text manifest (one absolute path per line). This manifest format maps directly onto pycmplot's list-based input specification. The column-wise merge required by CMplot was performed as part of its timed benchmark run (see S5.3).

S4. Computational Environment

S4.1 HPC environment

All benchmarks were executed on a single compute node of Rockfish (https://docs.arch.jhu.edu/en/latest/1_Clusters/Rockfish/), a SLURM-managed HPC cluster at Johns Hopkins University. Each benchmark job was allocated:

- CPU cores: 1 (*--cpus-per-task=1*)
- Memory: 64 GB
- Wall-time limit: 12 hours
- Node CPU: Intel Xeon Gold 6248R (Cascade Lake)
- Node RAM: 192 GB DDR4 2933MHz

Jobs were submitted as independent SLURM arrays — one job per tool — to ensure complete process isolation between tools and prevent cross-contamination of timing and memory measurements.

S5. Benchmark Procedure

S5.1 Timing scope

Wall-clock time was measured across the full end-to-end pipeline for each tool: (i) data loading from disk, (ii) in-memory data preparation, (iii) plot rendering, and (iv) output file writing. This reflects the real user experience from raw summary statistics to saved figure. Timing was initiated immediately before data loading and terminated immediately after the output file handle was closed. Python tools used *time.perf_counter()*; R tools used *proc.time()[["elapsed"]]*.

S5.2 Experimental conditions

Thread counts were fixed at one across all tools by setting the following environment variables prior to each run: *OMP_NUM_THREADS=1*, *MKL_NUM_THREADS=1*, *OPENBLAS_NUM_THREADS=1*, *NUMEXPR_NUM_THREADS=1*, and *R_DATATABLE_NUM_THREADS=1*. This ensures a fair single-core comparison; CMplot and qqman are inherently single-threaded and cannot utilize additional cores regardless of availability.

Five independent replicates were run per (tool, dataset size, and plot type) combination. A full garbage collection cycle was performed before each replicate — *gc.collect()* for Python tools, *gc(full=TRUE)* for R tools — to reduce carry-over allocation effects from prior runs within the same session. All output figures were saved as PNG at 150 DPI.

Each tool was installed in a dedicated conda environment to avoid dependency conflicts. Software versions are reported in Table S1.

S5.3 Multi-track input format and timing

Input format comparison

pycmplot: Accepts a list of N separate file paths. No preprocessing required.

CMplot: Requires traits to be merged column-wise into a single data frame (SNP, CHR, BP, $Trait_1$, ..., $Trait_n$) before the plot call can be made.

For pycmplot, multi-trait benchmarks passed a list of N file paths directly to the plotting functions via the manifest file, with no preprocessing required on the user's part.

For CMplot, the N trait files were loaded and merged column-wise within the timed section of the benchmark, because this merge is a mandatory prerequisite imposed by CMplot's input format. Specifically, the first trait file was loaded to obtain the variant scaffold (SNP, CHR, and "BP), and the p-value columns from each subsequent trait file were appended by positional alignment (valid because all traits share an identical variant order by construction). When many variants are not shared across traits or summary statistics, the required insertion of "NA" values can substantially increase CMplot's memory footprint, imposing unnecessary overhead in real-world applications. Reported wall-clock times for CMplot multi-track runs therefore reflect the total user-facing workflow cost, including data preparation.

S5.4 Memory Measurement

Peak memory for Python tools was measured using Python's *tracemalloc* module, which reports the peak traced heap allocation between *tracemalloc.start()* and *tracemalloc.stop()* calls surrounding each replicate.

Peak memory for R tools was measured using the Maximum Resident Set Size (MaxRSS) reported by */usr/bin/time -v*. Each R replicate was executed in an isolated *Rscript* process to ensure per-replicate MaxRSS values, since R's garbage collector does not reliably return memory to the OS between replicates within a single session.

S5.5 Output file size

The size of each generated PNG figure was recorded in kilobytes as a secondary metric, reflecting rendering complexity and the volume of plotted features (e.g., number of significant loci annotated) across tools and dataset sizes.

S6. Statistical Analysis

For each (tool, dataset size, plot type) combination, mean and standard deviation were calculated across five replicates. Outlier replicates were not excluded; high variance in a tool's performance is itself a relevant and reported characteristic (notably observed in CMplot due to R garbage collection timing variability). Speedup ratios were computed as:

$$Speedup = t_{\mathrm{CMplot}} / t_{\mathrm{tool}}$$

where t denotes the mean wall-clock time across five replicates at matched dataset size and plot type. Speedup ratios greater than 1 indicate the tool is faster than CMplot. All statistical summaries and speedup tables were generated using the provided *collect_results.py* script.

S7. Code Availability

All benchmark scripts, figures, and summary tables are available in the *benchmark* directory of the pycmplot GitHub repository at https://github.com/esohkevin/pycmplot.

Input data can be regenerated deterministically from the scripts using the fixed random seeds specified above (no data redistribution required). Software environment specifications (conda environment YAML files) are provided alongside the benchmark scripts.

S8. Nearest-gene Annotation of GWAS Lead SNPs

For each set of summary statistics, pycmplot first reduces the table of significant hits produced by the data load step to one representative SNP per locus using a greedy distance-based clumping procedure; i) within every chromosome, candidate leads are sorted from most to least significant, ii) within windows of w kilobases, the most significant SNP is selected and the rest discarded, iii) the next significant SNP is selected from the next window only if it lies more than w kilobases from every already-accepted lead. At the moment, a default window_kb = 500 kb is hardcoded in pycmplot but will be replaced by linkage disequilibrium (LD) clumping in the future. A gene reference table is constructed once per run from a lightweight Ensembl GFF3 TSV included in the package (in both hg38 and hg19 coordinates). The reference is pre-processed into a chromosome-keyed structure that stores, for each contig, the tuple list [(START, END, STRAND, GENE, BIOTYPE)] sorted by start position and a parallel flat array of start coordinates. The flat array serves as the sorted list used by the bisect_left python module to find the genes around where each lead SNP falls, terminating the search as soon as

start > pos + window_kb. This ensures that an increase in the number of lead SNPs does not substantially increase the lookup time. During the lookup, pycmplot stores a list of all the genes within the clumped lead SNP window as well as the number of genes in this window (*gene_density*) which is reported in the "hits summary" table. If there are multiple candidate genes, pycmplot then proceeds to select the best candidate(s) as shown in SFigure 6:

i) Lead SNP falls within one gene body (start ≤ POS ≤ end): The gene is selected (*nearest_gene*), the position is genic (*genic = True*), and the search is terminated.

ii) Window contains one gene and lead SNP is outside gene body: The position is non-genic (*genic = False*) and the gene is selected (*nearest_gene*).

iii) Window contains multiple genes with multiple genic and multiple non-genic: pycmplot prioritizes the best candidate (*nearest_gene*) based on a priority score calculated as shown in the figure. Briefly, pycmplot ensures that genic candidates are prioritized over non-genic candidates by awarding genic candidates a score of 2. If multiple genic candidates exist, they are then discriminated based on a promoter flag (a strand-aware check for whether lead SNP is within 2 kb of the gene transcription start site - TSS), and their biotype proximity score which is a product of the biotype weights and a weighted distance score. Biotypes were obtained from the Ensembl website (https://jun2026.archive.ensembl.org/info/genome/genebuild/biotypes.html) and weights were assigned based on the described biological role.

    If the window contains multiple non-genic candidates and all candidates fall on one side of the lead SNP, selection falls back to (ii) above using only distance to select the *nearest_gene.* Otherwise:

iv) All genes for which POS < START (that is the lead SNP falls at a lower coordinate than the gene start position in the gene's orientation – meaning the gene is downstream of the lead SNP), the gene with smallest distance is selected as *nearest_downstream_gene*.

v) For all genes whose END < POS (end position of the gene falls at a lower coordinate than the lead SNP in the gene's orientation – meaning lead SNP is downstream of the gene or the gene is upstream of the lead SNP), the gene with smallest distance is selected as *nearest_upstream_gene*. The two candidates in (iv) and (v) are joined to populate the *top_gene* field.

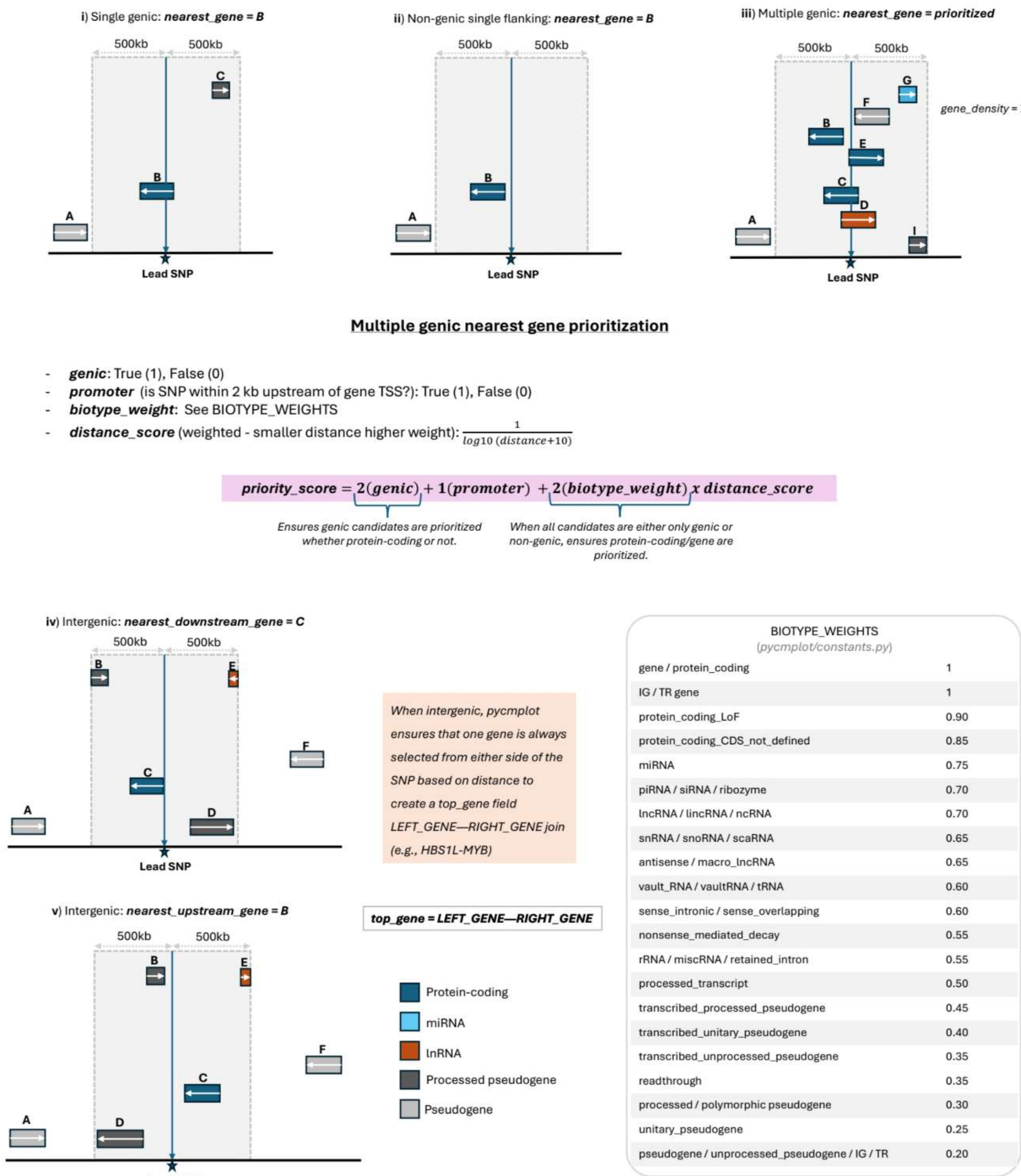

SFigure 1. Gene selection for annotation in pycmplot

The resulting per-lead record (SNP, CHR, POS, P, LABEL, biotype, top_gene, nearest_gene, nearest_gene_distance, nearest_upstream_gene, upstream_distance, nearest_downstream_gene, downstream_distance, promoter_upstream_flag, gene_density, priority_score) becomes one row of the hits summary table written to disk.

How does pycmplot select which column to annotate by?

If the lead SNP is genic, pycmplot annotates by '*nearest_gene*'. Otherwise '*top_gene*' is used for annotation. However, users can specify which column to annotate by using the "--*annotate <column>*" option via the CLI or "*annotate=<columne>*" option or the "*annotate='gene'*" + "*label_col=<column>*" combination via the Python API. In addition, pycmplot saves the hits table as a user-editable overlay (*hits.<group-key>.tsv*) when the cache option is invoked. This table can be edited to customize visualizations as shown in the examples.

S9. More Examples

Multi-panel GWAS of fetal hemoglobin (HbF) from the Wonkam et al., study (Wonkam *et al.* 2025) plotting on one axis . The study involved the utilization of multiple reference haplotype panels for genotype imputation and association analysis using data from all panels, rather than selecting a "best-performing" panel. Significant signals observed in specific panels or shared across the panels can now be represented compactly in one figure using *pycmplot*, allowing direct visual comparison. These are demonstrated in figures SFigure 2 through SFigure 5. SFigure 6 and SFigure 7 demonstration signed selection statistics. The commands used to generate the figures are also shown.

Circular without build information

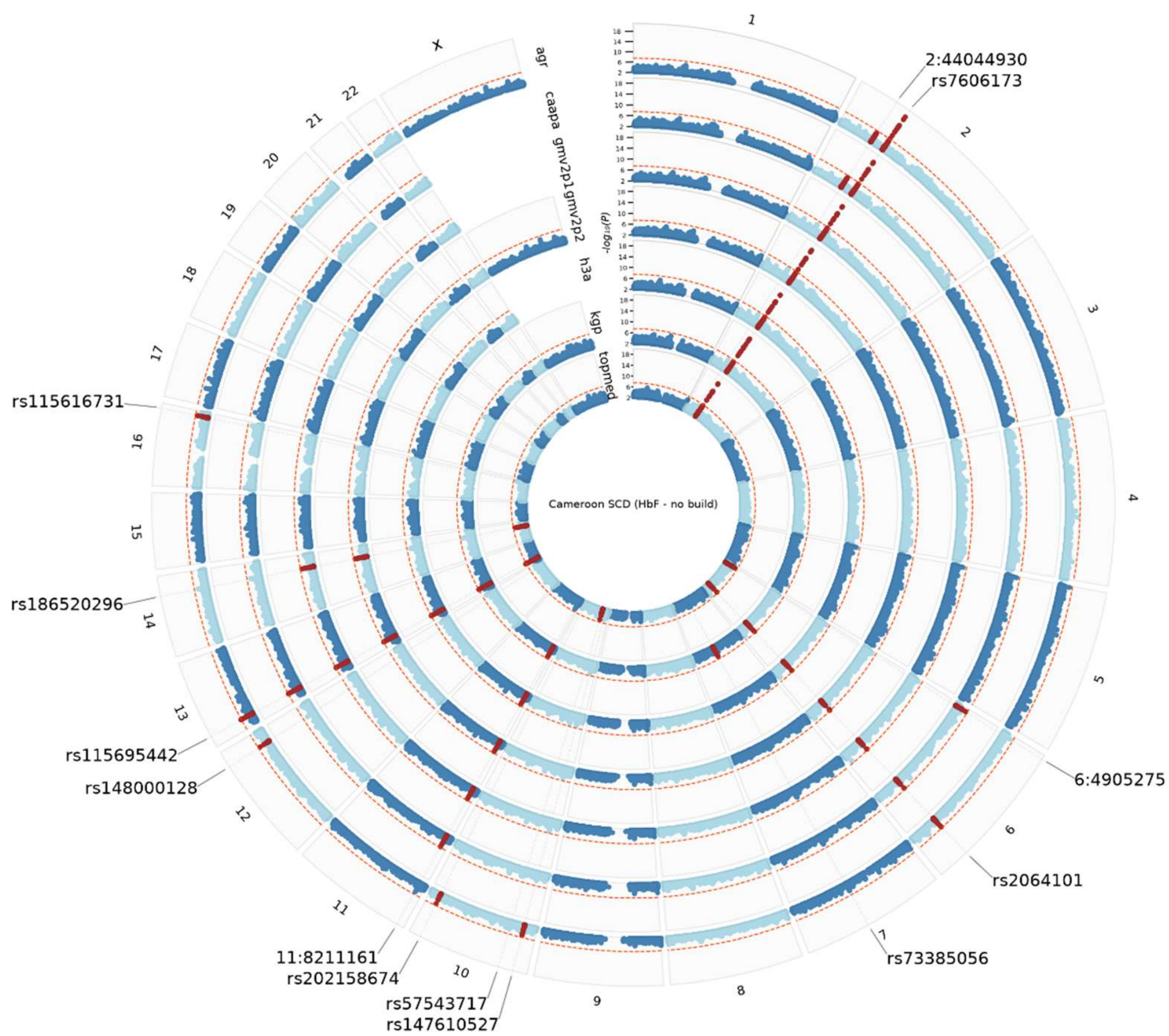

SFigure 2. Circular multi-track Manhattan plots of HbF GWAS in SCD in Cameroon without *build* information specified.

Each track represents association results from a different imputation panel (agr, caapa, gmv2p1, gmv2p2, h3a, kgp, and topmed). Significant loci are annotated. Without *build* information explicitly specified, pycmplot (> v0.2.8) will not perform gene lookup and will revert to annotate significant hits by SNP ID even if gene annotation is specified. Gene annotation is only performed if the *build* information of each summary statistic file is explicitly specified (see below).

Code

```
# Circular – with no build information

pycmplot \
  --mode cm \
  --sum_stats /data/awonkam1/kesoh/gwas/cmr/sumher/agr/nHbF/agr-r2-0.60-maf-0.01_nHbF_agecut-
5_kvik1.step2.assoc.gz,/data/awonkam1/kesoh/gwas/cmr/sumher/caapa/nHbF/caapa-r2-0.60-maf-
0.01_nHbF_agecut-5_kvik1.step2.assoc.gz,/data/awonkam1/kesoh/gwas/cmr/sumher/gmv2p1/nHbF/gmv2p1-r2-
0.60-maf-0.01_nHbF_agecut-
5_kvik1.step2.assoc.gz,/data/awonkam1/kesoh/gwas/cmr/sumher/gmv2p2/nHbF/gmv2p2-r2-0.60-maf-
0.01_nHbF_agecut-5_kvik1.step2.assoc.gz,/data/awonkam1/kesoh/gwas/cmr/sumher/h3a/nHbF/h3a-r2-0.60-maf-
0.01_nHbF_agecut-5_kvik1.step2.assoc.gz,/data/awonkam1/kesoh/gwas/cmr/sumher/kgp/nHbF/kgp-r2-0.60-maf-
0.01_nHbF_agecut-5_kvik1.step2.assoc.gz,/data/awonkam1/kesoh/gwas/cmr/sumher/topmed/nHbF/topmed-r2-
0.60-maf-0.01_nHbF_agecut-5_kvik1.step2.assoc.gz \
  --labels agr,caapa,gmv2p1,gmv2p2,h3a,kgp,topmed \
  --logp \
  --annotation_size 8 \
  --signif_line 5e-08 \
  --signif_threshold 5e-7 \
  --highlight \
  --highlight_line \
  --highlight_line_color lightgrey \
  --highlight_thresh 5e-7 \
  --annotate GENE \
  --chrom_label_side outside \
  --trim_pval 0.01 \
  --colors lightblue,steelblue \
  -qq \
  -qq_thin \
  -thin_below 0.01 \
  --qq_overlay \
  --dpi 300 \
  --output_dir ../pycmplot-output \
  --plot_title "Cameroon SCD (HbF - no build)" \
  --min_radius 20 \
  --plot_title_size 5
```

Circular with build information

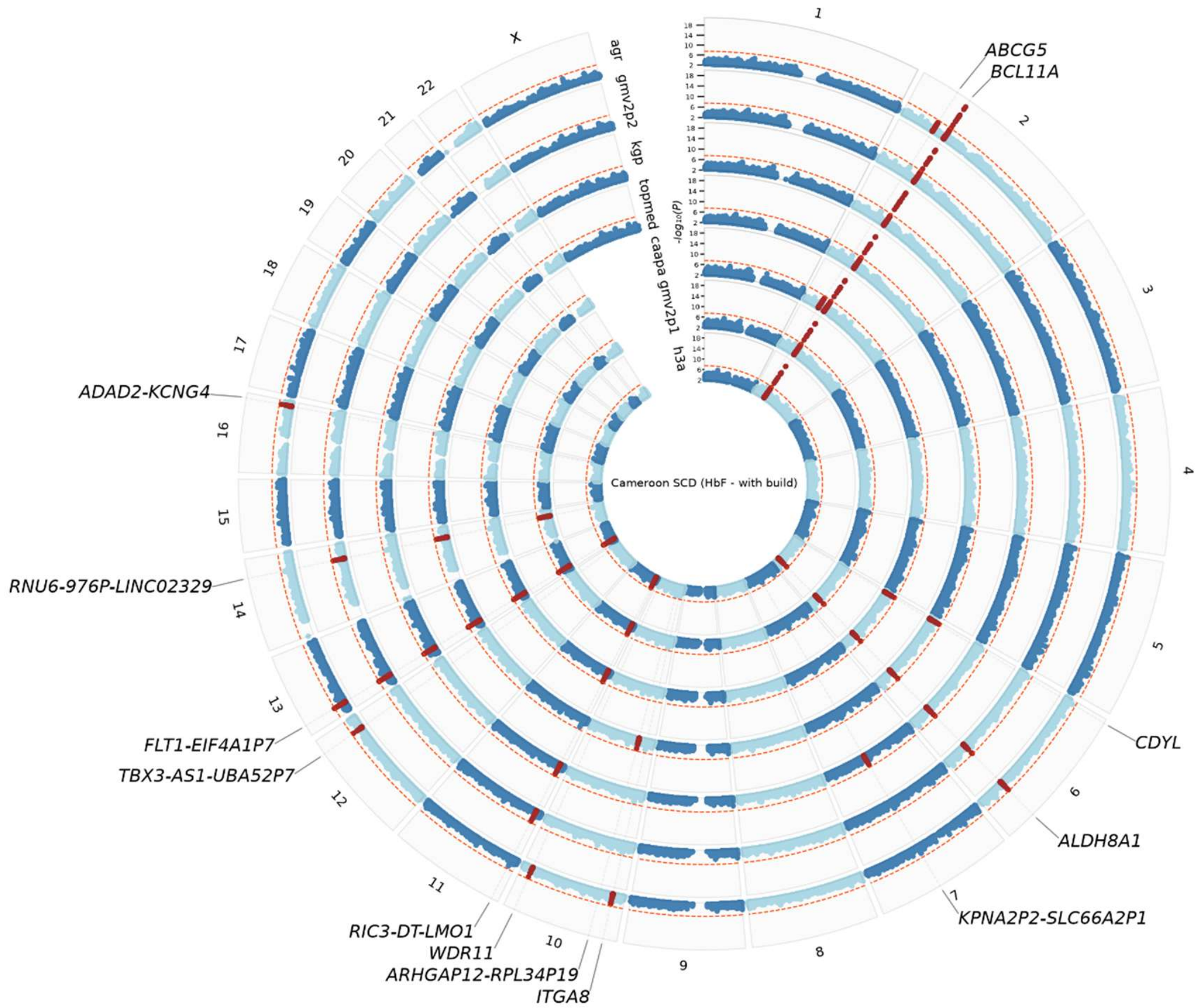

SFigure 3. Circular multi-track Manhattan plots of HbF GWAS in SCD in Cameroon with *build* information specified. Significant signals were annotated by the "top_gene" column of the hits table.

Significant loci shared by all the panels can be seen on chromosomes 2 *(BCL11A*), 6 (*ALDH8A1* downstream of the known *HBS1L-MYB* locus), and 13 (*FLT1*). Other loci shared by only some panels (*CDYL*, *RIC3-DT-LMO1*) or specific to some panels (e.g. *ABCG5, WDR11*) are visible. Unlike CMplot, pycmplot leaves space between the first and last chromosomes (sectors) to accommodate the y-axis label and track labels explicitly. The plot also contrasts support for the X chromosome by the different panels clearly. pycmplot includes a "*--sort_track chrom_len*" options than can allow the tracks to be sorted by number of chromosomes such that summary statistics files without the X chromosome are grouped together in the inner tracks.

Code

```
# Circular – with build information

pycmplot \
  --mode cm \
  --sum_stats /data/awonkam1/kesoh/gwas/cmr/sumher/agr/nHbF/agr-r2-0.60-maf-0.01_nHbF_agecut-
5_kvik1.step2.assoc.gz,/data/awonkam1/kesoh/gwas/cmr/sumher/caapa/nHbF/caapa-r2-0.60-maf-
0.01_nHbF_agecut-5_kvik1.step2.assoc.gz,/data/awonkam1/kesoh/gwas/cmr/sumher/gmv2p1/nHbF/gmv2p1-r2-
0.60-maf-0.01_nHbF_agecut-
5_kvik1.step2.assoc.gz,/data/awonkam1/kesoh/gwas/cmr/sumher/gmv2p2/nHbF/gmv2p2-r2-0.60-maf-
0.01_nHbF_agecut-5_kvik1.step2.assoc.gz,/data/awonkam1/kesoh/gwas/cmr/sumher/h3a/nHbF/h3a-r2-0.60-maf-
0.01_nHbF_agecut-5_kvik1.step2.assoc.gz,/data/awonkam1/kesoh/gwas/cmr/sumher/kgp/nHbF/kgp-r2-0.60-maf-
0.01_nHbF_agecut-5_kvik1.step2.assoc.gz,/data/awonkam1/kesoh/gwas/cmr/sumher/topmed/nHbF/topmed-r2-
0.60-maf-0.01_nHbF_agecut-5_kvik1.step2.assoc.gz \
  --labels agr,caapa,gmv2p1,gmv2p2,h3a,kgp,topmed \
  --build hg19,hg19,hg19,hg38,hg38,hg19,hg38 \
  --logp \
  --annotation_size 8 \
  --signif_line 5e-08 \
  --signif_threshold 5e-7 \
  --highlight \
  --highlight_line \
  --highlight_line_color lightgrey \
  --highlight_thresh 5e-7 \
  --annotate GENE \
  --chrom_label_side outside \
  --trim_pval 0.01 \
  --colors lightblue,steelblue \
  -qq \
  -qq_thin \
  -thin_below 0.01 \
  --qq_overlay \
  --dpi 300 \
  --output_dir ../pycmplot-output \
  --plot_title "Cameroon SCD (HbF - with build)" \
  --min_radius 20 \
  --plot_title_size 5 \
  --sort_track chrom_len
```

Linear with build information

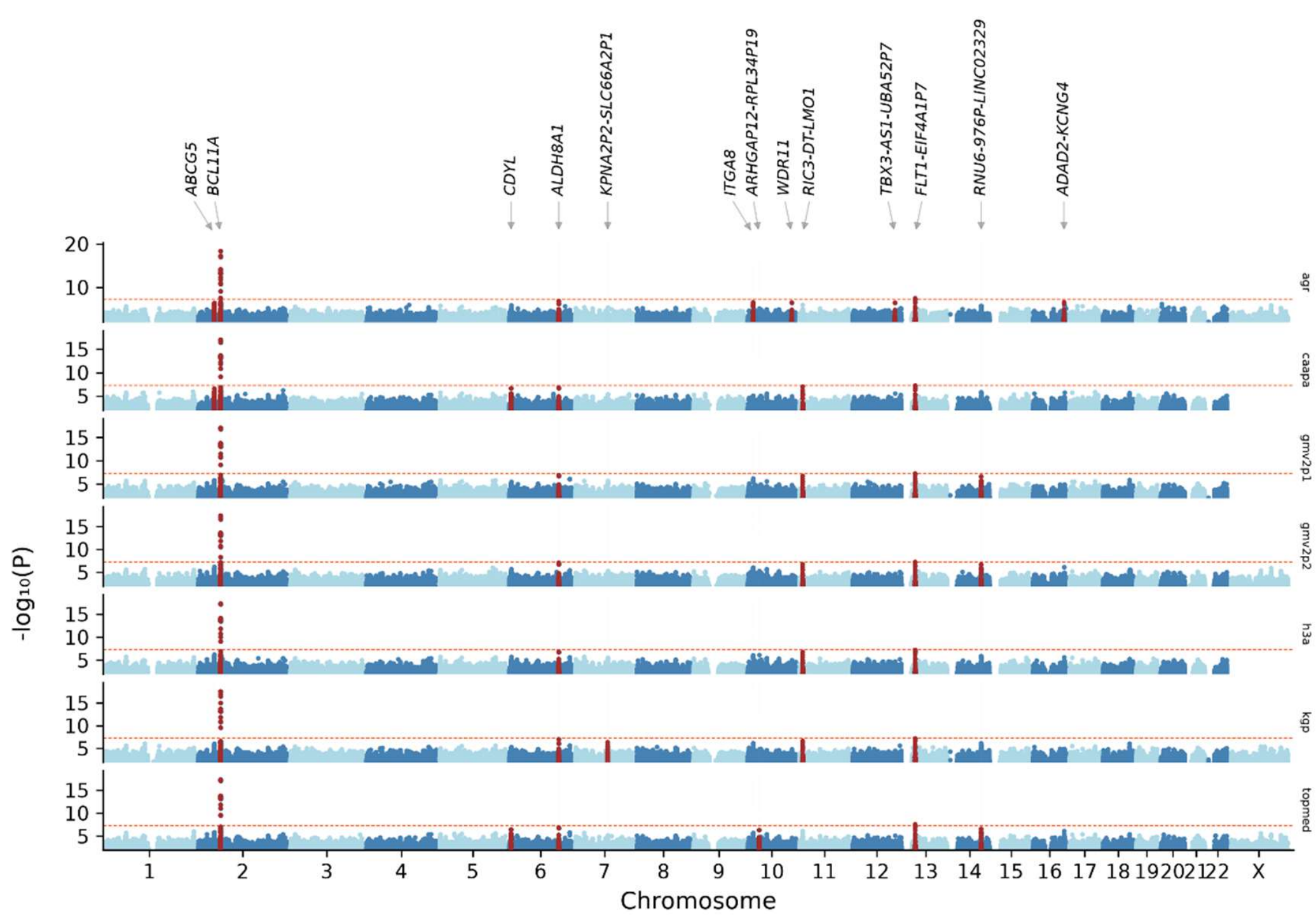

SFigure 4. Linear multi-track Manhattan plots of HbF GWAS in SCD in Cameroon.

The linear rendering presents the same visual contrast as the circular. It is fundamentally different from other multi-track/multi-trait visual rendering by other tools in that it provides a single annotation track at the top of the figure and draws lines through all tracks at highlighted positions to allow visual comparison of shared and track-specific significant loci.

Code

```
# Linear – with build information

pycmplot \
  --mode lm \
  --sum_stats /data/awonkam1/kesoh/gwas/cmr/sumher/agr/nHbF/agr-r2-0.60-maf-0.01_nHbF_agecut-
5_kvik1.step2.assoc.gz,/data/awonkam1/kesoh/gwas/cmr/sumher/caapa/nHbF/caapa-r2-0.60-maf-
0.01_nHbF_agecut-5_kvik1.step2.assoc.gz,/data/awonkam1/kesoh/gwas/cmr/sumher/gmv2p1/nHbF/gmv2p1-r2-
0.60-maf-0.01_nHbF_agecut-
5_kvik1.step2.assoc.gz,/data/awonkam1/kesoh/gwas/cmr/sumher/gmv2p2/nHbF/gmv2p2-r2-0.60-maf-
0.01_nHbF_agecut-5_kvik1.step2.assoc.gz,/data/awonkam1/kesoh/gwas/cmr/sumher/h3a/nHbF/h3a-r2-0.60-maf-
0.01_nHbF_agecut-5_kvik1.step2.assoc.gz,/data/awonkam1/kesoh/gwas/cmr/sumher/kgp/nHbF/kgp-r2-0.60-maf-
0.01_nHbF_agecut-5_kvik1.step2.assoc.gz,/data/awonkam1/kesoh/gwas/cmr/sumher/topmed/nHbF/topmed-r2-
0.60-maf-0.01_nHbF_agecut-5_kvik1.step2.assoc.gz \
  --labels agr,caapa,gmv2p1,gmv2p2,h3a,kgp,topmed \
  --build hg19,hg19,hg19,hg38,hg38,hg19,hg38 \
  --logp \
  --annotation_size 8 \
  --signif_line 5e-08 \
  --signif_threshold 5e-7 \
  --highlight \
  --highlight_line \
  --highlight_line_color lightgrey \
  --highlight_thresh 5e-7 \
  --annotate GENE \
  --trim_pval 0.01 \
  --colors lightblue,steelblue \
  -qq \
  -qq_thin \
  -thin_below 0.01 \
  --qq_overlay \
  --dpi 300 \
  --output_dir ../pycmplot-output \
  --plot_title "Cameroon SCD (HbF)" \
  --plot_title_size 7 \
  --track_heights 1.5,1,1,1,1,1,1,1 \
  --figure_size 10,7
```

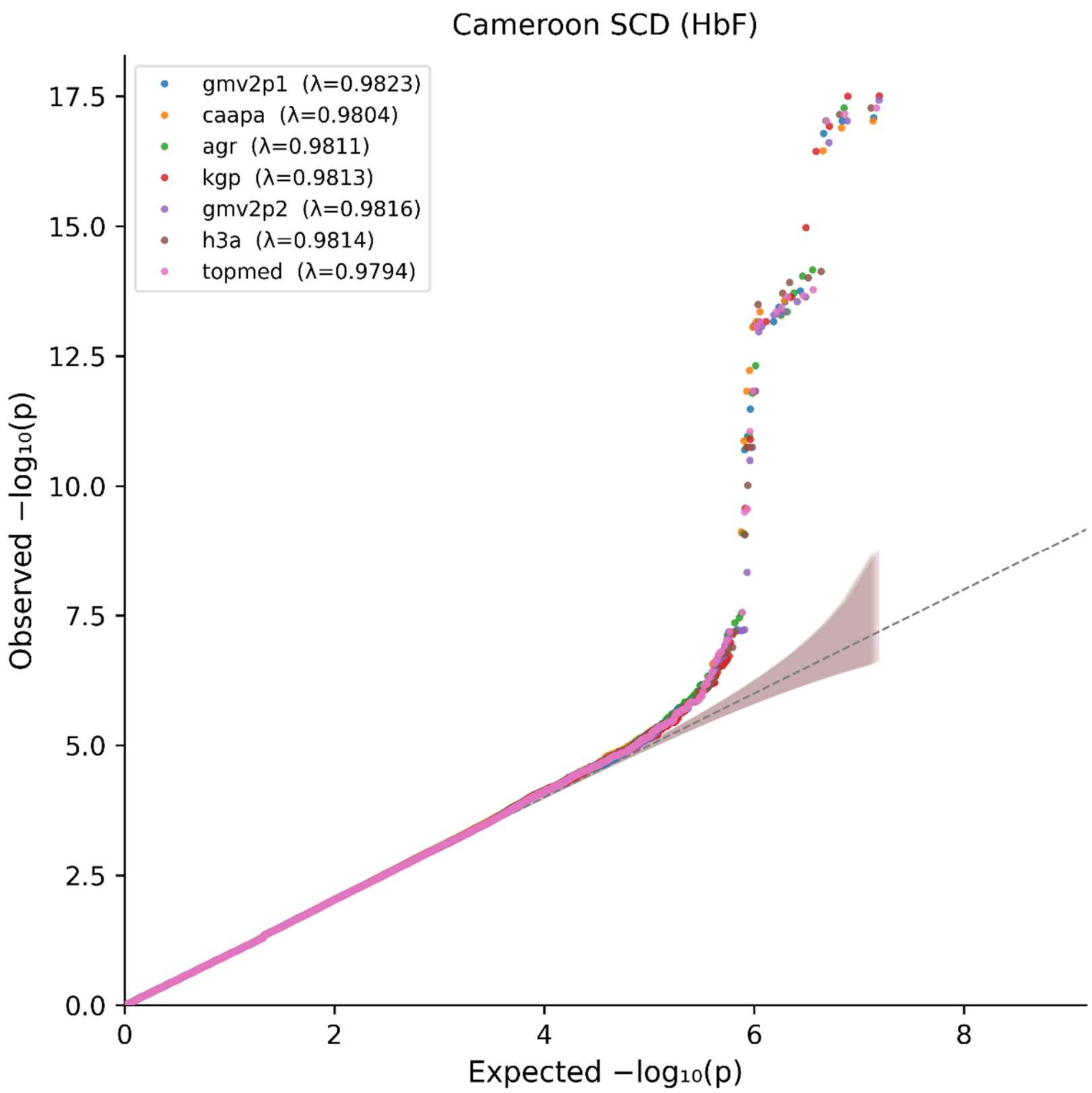

SFigure 5. Overlay qq-plots of HbF GWAS in SCD in Cameroon.

QQ-plots are generated when the circular and linear plots above were generated because a `-*qq*` option was specified.

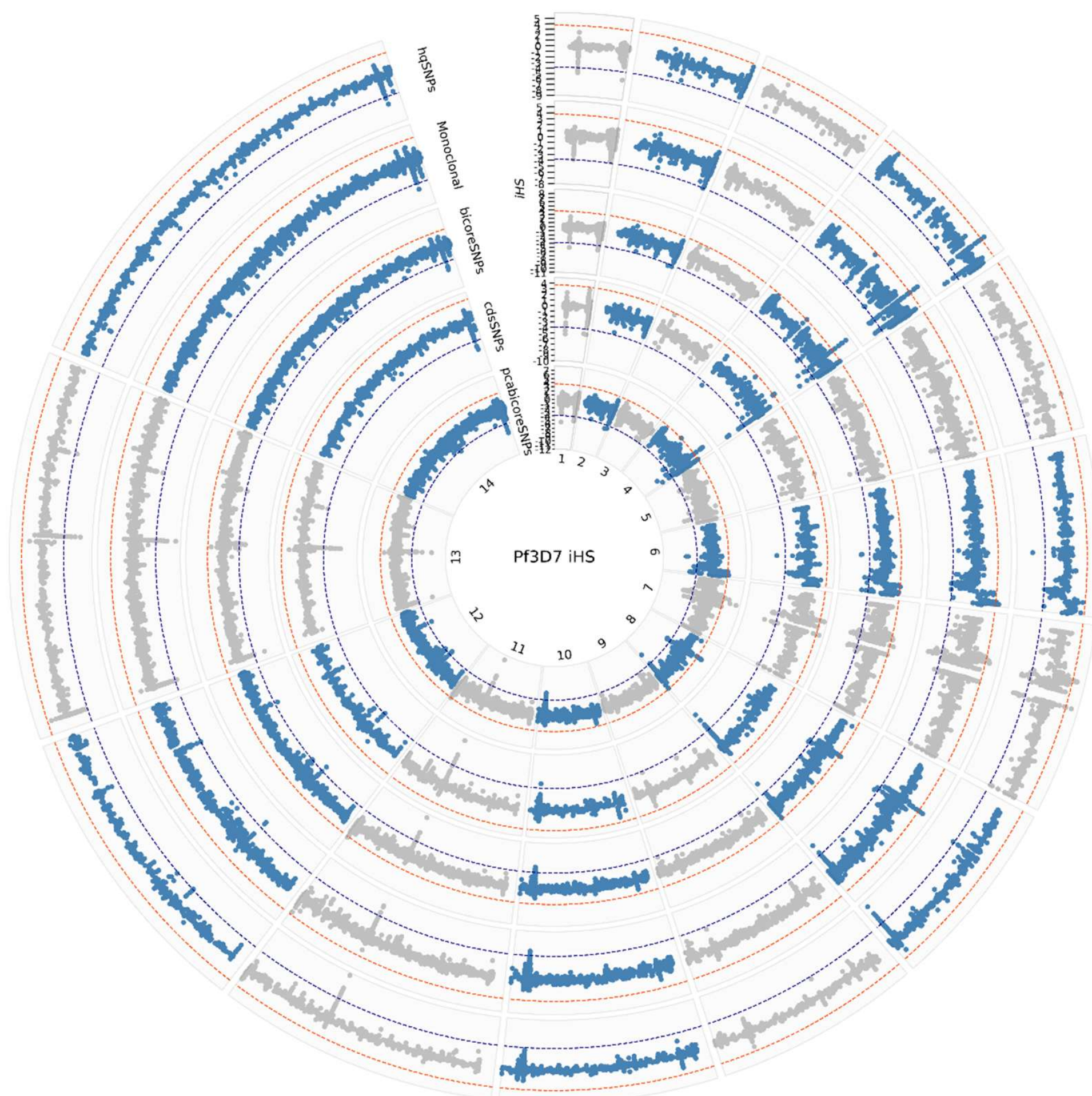

SFigure 6. Circular multi-track Manhattan plots of integrated haplotype score (iHS)

Circular genome-wide plots of integrated haplotype score (iHS) statistics from *Plasmodium falciparum* populations sampled in Cameroon (unpublished work).

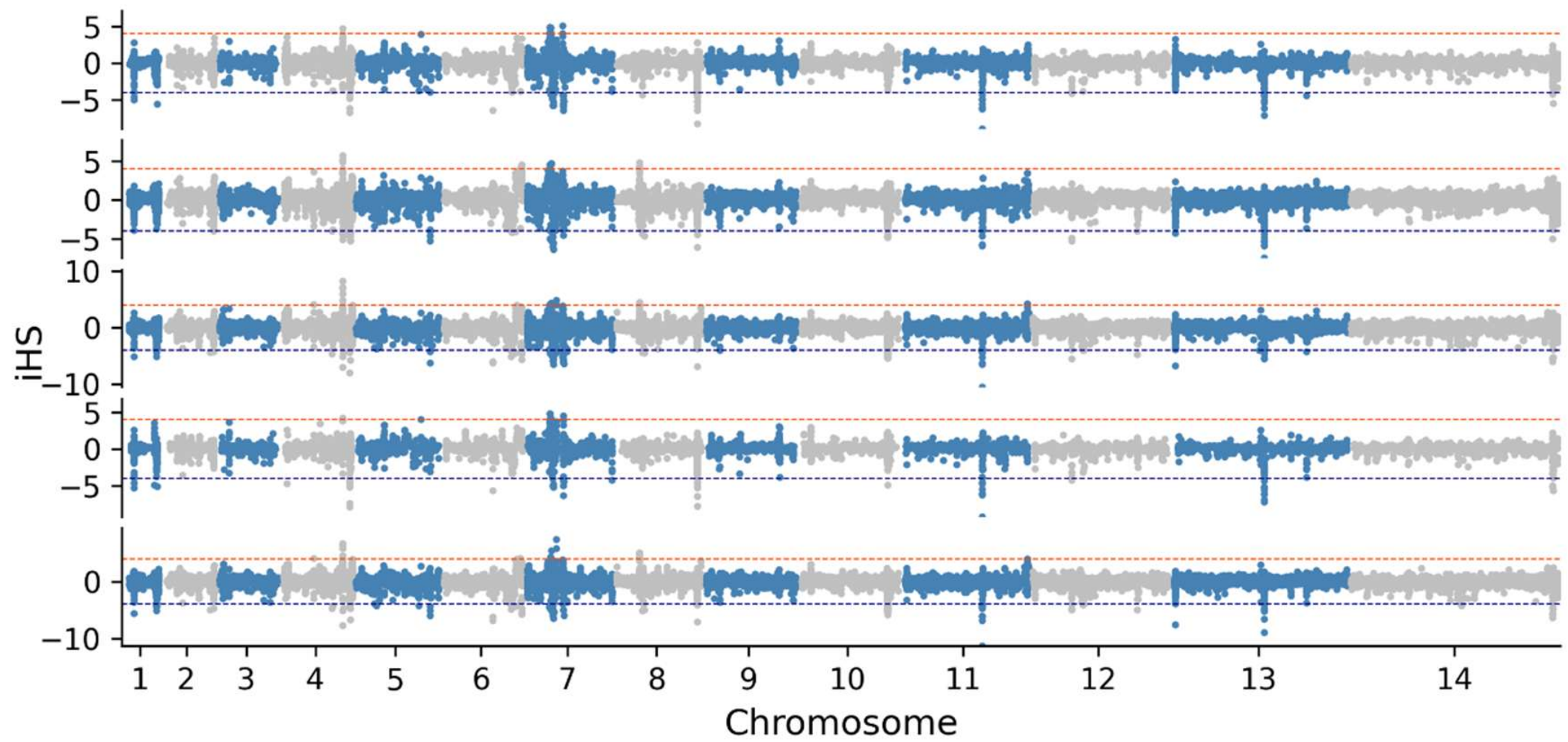

SFigure 7. Linear multi-track Manhattan plots of integrated haplotype score (iHS)

Linear genome-wide plots of integrated haplotype score (iHS) statistics from *Plasmodium falciparum* populations sampled in Cameroon.

SFigure 6 and SFigure 7 demonstrate support for population genetic selection scans and non-GWAS genomic summary statistics. These examples highlight pycmplot's ability to efficiently visualize heterogeneous genome-wide datasets using both circular and linear layouts, while supporting multiple tracks, flexible annotation, and large-scale genomic data exploration.

Commands used to generate SFigure 6 and 7

```
# Circular Manhattan
pycmplot \
        --mode cm \
        --sum_stats hqcoreiHSresult.txt,monoclonaliHSresult.txt,bipcoreiHSresult.txt,cdsiHSresult.txt,pcabicopassiHSresult.txt \
        --labels hqSNPs,Monoclonal,bicoreSNPs,cdsSNPs,pcabicoreSNPs \
        --pval_column iHS \
        --pos_column POSITION \
        --snp_column SNP \
        --chrom_column CHR \
        --ylabel iHS \
        --plot_title "Pf3D7 iHS"\
         --signif_line 4 \
        --suggest_threshold 4 \
        --dpi 300
```

```
# Linear Manhattan
pycmplot \
	--mode lm \
	--sum_stats hqcoreiHSresult.txt,monoclonaliHSresult.txt,bipcoreiHSresult.txt,cdsiHSresult.txt,pcabicopassiHSresult.txt \
	--labels hqSNPs,Monoclonal,bicoreSNPs,cdsSNPs,pcabicoreSNPs \
	--pval_column iHS \
	--pos_column POSITION \
	--snp_column SNP \
	--chrom_column CHR \
	--chr_spacing 0 \
	--figure_size 8,4 \
	--ylabel iHS \
	--plot_title "Pf3D7 iHS"\
	 --signif_line 4 \
	--suggest_threshold 4 \
	--dpi 300
```

A tutorial describing other use cases with additional pycmplot features are detailed in the documentation page at https://pycmplot.readthedocs.io/en/latest/cli_tutorial.html.

Python API code used to generate Figure 2 in the main text

```
import pycmplot as pcm

sumstats_list = [
    '/data/awonkam1/kesoh/gwas/cmr/sumher/agr/nHbF/agr-r2-0.60-maf-0.01_nHbF_agecut-5_kvik1.step2.assoc.gz',
    '/data/awonkam1/kesoh/gwas/cmr/sumher/caapa/nHbF/caapa-r2-0.60-maf-0.01_nHbF_agecut-5_kvik1.step2.assoc.gz',
    '/data/awonkam1/kesoh/gwas/cmr/sumher/gmv2p1/nHbF/gmv2p1-r2-0.60-maf-0.01_nHbF_agecut-5_kvik1.step2.assoc.gz',
    '/data/awonkam1/kesoh/gwas/cmr/sumher/gmv2p2/nHbF/gmv2p2-r2-0.60-maf-0.01_nHbF_agecut-5_kvik1.step2.assoc.gz',
    '/data/awonkam1/kesoh/gwas/cmr/sumher/h3a/nHbF/h3a-r2-0.60-maf-0.01_nHbF_agecut-5_kvik1.step2.assoc.gz',
    '/data/awonkam1/kesoh/gwas/cmr/sumher/kgp/nHbF/kgp-r2-0.60-maf-0.01_nHbF_agecut-5_kvik1.step2.assoc.gz',
    '/data/awonkam1/kesoh/gwas/cmr/sumher/topmed/nHbF/topmed-r2-0.60-maf-0.01_nHbF_agecut-5_kvik1.step2.assoc.gz'
]

label_list = ['agr','caapa','gmv2p1','gmv2p2','h3a','kgp','topmed']

build_list = ['hg19','hg19','hg19','hg38','hg38','hg19','hg38']


# continued below
```

```
# LOADING THE SUMMARY STATS
################################################################
# No Build
info_no_build = pcm.prep(
    sum_stats=sumstats_list,
    labels=label_list
)

loaded_no_build = pcm.load(
    sum_stats=sumstats_list,
    labels=label_list,
    file_info=info_no_build,
    highlight=True,
    highlight_thresh=5e-7,
    signif_threshold=5e-7,
    signif_line=True,
    logp=True,
    table_out="multi_panel_no_build",
    trim_pval=0.01,
    cache=True,
    cache_dir="/scratch4/awonkam1/kesoh/gwas/pycmplotbenchmark/cache"
)


################################################################
# With Build
info_with_build = pcm.prep(
    sum_stats=sumstats_list,
    labels=label_list,
    build_list=build_list
)

loaded_with_build = pcm.load(
    sum_stats=sumstats_list,
    highlight=True,
    labels=label_list,
    file_info=info_with_build,
    logp=True,
    table_out="multi_panel_with_build",
    trim_pval=0.001,
    signif_threshold=5e-7,
    highlight_thresh=5e-7,
    sort_tracks="chrom_len",
    cache=True,
    cache_dir="/scratch4/awonkam1/kesoh/gwas/pycmplotbenchmark/cache"
)

# continued below
```

```
import matplotlib.pyplot as plt
import pandas as pd
import numpy as np

# INITIALIZE A FIGURE FOR PLOTTING
#####################################

# Create subplots with projection='polar'
fig, ((ax1, ax2), (ax3, ax4)) = plt.subplots(
    2, 2,
    figsize=(14, 14),
    subplot_kw={'projection': 'polar'},
    gridspec_kw={
        'hspace': 0.1,
        'wspace': 0.1
    }
)

# continued below
```

```
# PLOTTING WITH UNEDITED HITS TABLE
####################################

# No build
f1 = pcm.circular(
    sumstats_loaded=loaded_no_build["dfs"],
    hits_table=loaded_no_build["annot"],
    sector_sizes=loaded_no_build["sectors"],
    signif_lines=loaded_no_build["lines"],
    signif_line=True, suggest_line=True, signif_threshold=5e-8,
    annotate="GENE",
    plot_title="No Build",
    plot_title_size=7,
    output_format='png',
    annotation_size=8,
    track_label_size=5,
    dpi=300,
    highlight=True,
    highlight_line=True, highlight_thresh=5e-8,
    chrom_label_side='outside',
    pad=1,
    r_min=20,
    r_max=100,
    logp=True,
    ax=ax1, # Pass subfigure 1
)

# With build
f2 = pcm.circular(
    sumstats_loaded=loaded_with_build["dfs"],
    hits_table=loaded_with_build["annot"],
    sector_sizes=loaded_with_build["sectors"],
    signif_lines=loaded_with_build["lines"],
    signif_line=True, suggest_line=True, signif_threshold=5e-7,
    annotate='gene',
    annotation_size=8,
    track_label_size=5,
    label_col='top_gene',
    dpi=300,
    chrom_label_side='outside',
    logp=True,
    highlight=True,
    highlight_line=True, highlight_thresh=5e-7,
    plot_title="Build Specified",
    plot_title_size=7,
    r_min=20,
    colors=['steelblue','orange'],
    ax=ax2,  # Pass subfigure 2
)


# continued below
```

```
# PLOTTING WITH EDITED HITS TABLE
#####################################

# Edit the hits table to color different categories
hits = pd.read_csv("/scratch4/awonkam1/kesoh/gwas/pycmplotbenchmark/cache/annotations/hits.3c311cf6.tsv", sep="\t",
comment="#")

# Categories
hits['category'] = np.where(
    hits['P'] < 5e-8, "novel significant (P < 5e-8)",
    np.where(
        hits['P'] < 5e-7, "novel marginally significant (P < 5e-7)",
        "suggestive (P > 5e-7)"
    )
)

# Colors
hits['highlight_color'] = np.where(
    hits['P'] < 5e-8, "brown",
    np.where(
        hits['P'] < 5e-7, "orange",
        "grey"
    )
)

# Specific known and novel genes
hits['category'] = np.where(
    hits['nearest_gene'] == "BCL11A", "known significant (P < 5e-8)",
    np.where(
        hits['nearest_gene'] == "ALDH8A1", "known significant (P < 5e-8)",
        hits['category']
    )
)

hits['highlight_color'] = np.where(
    hits['nearest_gene'] == "BCL11A", "green",
    np.where(
        hits['nearest_gene'] == "ALDH8A1", "green",
        hits['highlight_color']
    )
)


# continued below
```

```
# With build - colored categories
f3 = pcm.circular(
    sumstats_loaded=loaded_with_build["dfs"],
    hits_table=hits,                          # <------- updated hits table
    sector_sizes=loaded_with_build["sectors"],
    signif_lines=loaded_with_build["lines"],
    signif_line=True, suggest_line=True, signif_threshold=5e-7,
    annotate="GENE", label_col="nearest_gene",
    plot_title="Build Specified",
    plot_title_size=7,
    output_format='png',
    annotation_size=8,
    track_label_size=5,
    dpi=300,
    highlight=True,
    highlight_line=True, highlight_thresh=5e-7,
    highlight_legend=True, highlight_legend_loc="upper left",
    chrom_label_side='outside',
    pad=1,
    r_min=20,
    r_max=100,
    logp=True,
    colors=['steelblue','lightblue'],
    ax=ax3, # Pass subfigure 3
)

# With build - marginal annotated
f4 = pcm.circular(
    sumstats_loaded=loaded_with_build["dfs"],
    hits_table=hits,                          # <------- updated hits table
    sector_sizes=loaded_with_build["sectors"],
    signif_lines=loaded_with_build["lines"],
    signif_line=True, suggest_line=True, signif_threshold=5e-8,
    annotate='gene',
    annotation_size=8,
    track_label_size=5,
    label_col='nearest_gene',
    dpi=300,
    chrom_label_side='outside',
    logp=True,
    highlight=True, highlight_thresh=5e-8,
    highlight_line=True,
    highlight_legend=False,
    plot_title="Build Specified",
    plot_title_size=7,
    r_min=20,
    colors=['steelblue','lightblue'],
    ax=ax4,  # Pass subfigure 4
)

ax1.set_title("A", fontdict={'size': 9, 'fontweight': "bold"}, loc='left', pad=2)
ax2.set_title("B", fontdict={'size': 9, 'fontweight': "bold"}, loc='left', pad=2)
ax3.set_title("C", fontdict={'size': 9, 'fontweight': "bold"}, loc='left', pad=-2)
ax4.set_title("D", fontdict={'size': 9, 'fontweight': "bold"}, loc='left', pad=2)


# END
```

References

D. Turner S. qqman: an R package for visualizing GWAS results using Q-Q and manhattan plots. *J Open Source Softw* 2018;**3**(25):731. https://doi.org/10.21105/joss.00731.

He Y, Koido M, Shimmori Y *et al. GWASLab: A Python Package for Processing and Visualizing GWAS Summary Statistics*. published online 1 May 2023. https://doi.org/10.51094/jxiv.370.

Wonkam A, Esoh K, Levine RM *et al.* FLT1 and other candidate fetal haemoglobin modifying loci in sickle cell disease in African ancestries. *Nat Commun* 2025;**16**(1):2092. https://doi.org/10.1038/s41467-025-57413-5.

Yin L, Zhang H, Tang Z *et al.* rMVP: A Memory-Efficient, Visualization-Enhanced, and Parallel-Accelerated Tool for Genome-Wide Association Study. *Genomics Proteomics Bioinformatics* 2021;**19**(4):619–28. https://doi.org/10.1016/j.gpb.2020.10.007.
